# Evaluating impact of Nd: YAG laser associated defects on optical quality of hydrophilic and hydrophobic intraocular lenses using visualization of light propagation and USAF test targets

**DOI:** 10.1186/s12886-022-02738-8

**Published:** 2022-12-16

**Authors:** A. F. Borkenstein, E. M. Borkenstein, P. Omidi, A. Langenbucher

**Affiliations:** 1 Borkenstein & Borkenstein private practice, Privatklinik der Kreuzschwestern Graz, Kreuzgasse 35, 8010 Graz, Austria; 2 Institut für Experimentelle Ophthalmologie, Universität des Saarlandes, Saar 66424 Homburg, Deutschland

**Keywords:** Defects in intraocular lenses, YAG-pits, Visualization of light propagation, USAF test targets, Optical quality

## Abstract

**Background:**

Neodymium:yttrium aluminum garnet (Nd:YAG) laser capsulotomy is a well-accepted, safe, and effective measure in the treatment of posterior capsule opacification. However, iatrogenic intraocular lens damage is a relatively common side effect that happens due to inappropriate focusing during the procedure. This experimental study analyzes the impact of YAG-pits to obtain qualitative information.

**Methods:**

Acrylic, monofocal hydrophilic and hydrophobic intraocular lenses (IOLs) with 6.0 mm optic and the with the same power (21D) were studied. First, all measurements were done with unmodified IOLs. Damage was intentionally created, performing YAG-pits (*n* = 5) in the central area of the lens optic (3.0 mm) using a photodisruption laser with the same energy level of 1.8 mJ. To simulate the cruciate pattern, the 5 defects were created in a cross shape within the 3.0 mm optical zone. Afterwards, all laboratory measurements were repeated: These included the United States Air Force (USAF) resolution test chart to study the imaging performance of the IOL, light field measurements to show the course of the rays behind the IOL and the modulation transfer function (MTF) measurements were analyzed.

**Results:**

Evaluating USAF showed that unmodified lenses produced a sharper image. Damaged lenses led to a more blurred image and to the impression of a lower contrast with a kind of halo/glare effect. The light field measurement showed that YAG-pits led to a kind of dispersion and scattering effect, which was higher in hydrophobic IOLs. MTF showed a deterioration in damaged hydrophilic and hydrophobic IOLs, respectively.

**Conclusion:**

Our experimental study confirms that YAG-pits can reduce imaging quality of intraocular lenses. These defects behave as a new Huygens source, distribute a spherical wave that additionally illuminate the background of the USAF target. It can be assumed that material properties of the IOL (water content, refractive index) play an important role and affect results. The impact level is strongly dependent on the number, size and position of YAG-pits within the optic. Limitation: Only monofocal IOLs have been investigated so far, further tests with various IOL optics have to follow. In addition, simulating the circular pattern of YAG capsulotomy is necessary.

## Background

Posterior capsule opacification (PCO) remains the most common long-term postoperative complication of modern cataract surgery [1]. PCO can reduce visual acuity (VA), decrease contrast sensitivity, and increase retinal straylight [2]. Neodymium:yttrium aluminum garnet (Nd:YAG) laser capsulotomy is a well-accepted, safe, and effective tool in the treatment of PCO [3, 4]. According to a real-world evidence study with > 20.000 eyes, the incidence of PCO ranges between 4.7 and 18.6% at 3 years and 7.1–22.6% at 5 years and the incidence of Nd:YAG capsulotomy ranges between 2.4 and 12.6% at 3 years and 5.8–19.3% at 5 years post-cataract surgery [5]. Even though the numbers for PCO and YAG capsulotomy are visibly high, one notices a wide variation. This is also due to the fact that different lens models and lens designs lead to different PCO rates and also because study designs and observation periods were chosen very differently in these evaluations.

YAG capsulotomy improves visual acuity and may also have positive effects on glare and contrast sensitivity in some cases. However, there are also reports on complications such as corneal injuries, pupillary block, iritis, intraocular pressure rises, vitreous prolapse, macular edema, retinal damage,IOL movement, IOL dislocation or impairment [6–9].

The purpose of this experimental study was to analyze the impact of YAG-pits in IOLs on the optical bench and to visualize the light propagation of monofocal hydrophilic and hydrophobic acrylic intraocular lenses with YAG-defects in order to obtain qualitative information on the image characteristics and also evaluate differences regarding lens material.

## Methods

Monofocal, one-piece IOLs with an optic diameter of 6.0 mm were divided into 2 groups according to the water content: Hydrophobic acrylic lenses (CT Lucia 611P, Zeiss Meditec, Germany) and hydrophilic acrylic lenses (Aspira-aA, HumanOptics, Germany) with the same power of 21.0D. The hydrophobic IOLs had a water content of 0.3% and a refractive index of 1.49, the hydrophilic IOLs had a water content of 26.0% and a refractive index of 1.46. First, all measurements were performed with unmodified lenses. Subsequently, the exact same measurements were performed with lenses that showed defects. In all samples, we created the same number of YAG-pits (*n* = 5) in the central part of the optics (3.0 mm), using a photodisruption laser (Visulas YAG III, Zeiss, Meditec) with the same energy levels of 1.8 mJ. The disruption laser (Laser class 4, IEC60825-1) is using a wavelength of 1064 nanometers (nm), a Super Gaussian mode and a pulse length of 2–3 nanoseconds (ns) and a focus diameter of 10 micrometers (µm). The focal point of the target beam was aimed directly at the posterior surface of the intraocular lens to create defects (YAG-pits) intentionally (focus shift 0 μm).

**Fig. 1:**
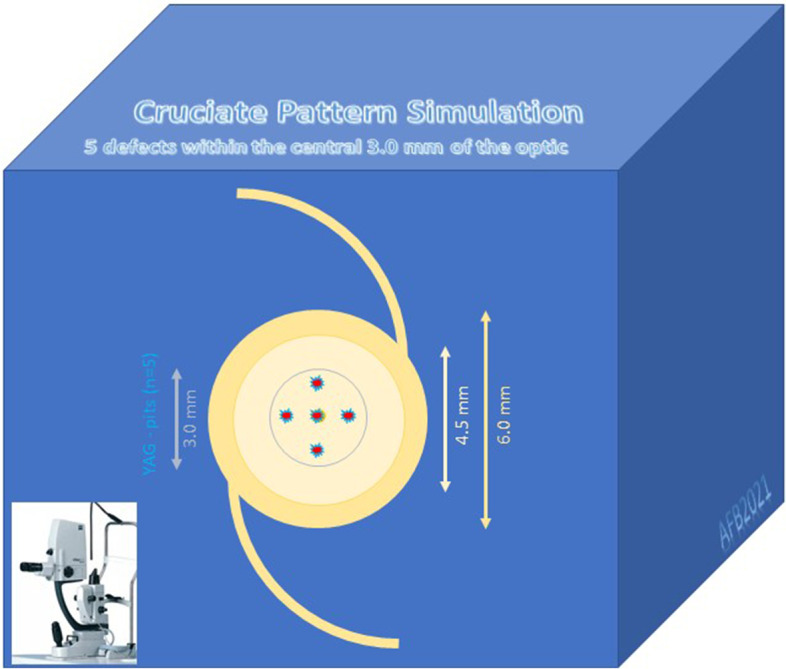
Laboratory test arrangement for simulating the “cruciate pattern”. The first defect was placed exactly in the center of the optics, the other 4 damages crosswise within the 3.0 mm zone, each with double distance to the next/neighbor defect. In all cases the same number of defects was created (*n* = 5) and the same laser settings (energy: 1.8 mJ) were used. The focal point of the target beam was aimed directly at the posterior surface of the optics

In our laboratory study creating the YAG-shots a “cruciate pattern” was chosen to simulate one of the most common techniques used in clinical routine. The first defect was set directly in the center of the optic, then 2 more defects were placed horizontally on the right and left side and 2 more defects were placed superiorly and inferiorly to the center within the 3.0 mm zone (Fig. 1). All measurements with unmodified lenses and lenses with defects were repeated 3 times (sample size of hydrophilic IOLs *n* = 3 and hydrophobic IOLs *n* = 3). Scanning Electron Microscopy was used to inspect and analyze the defects (YAG pits) and to measure them.

### USAF

**Fig. 2:**
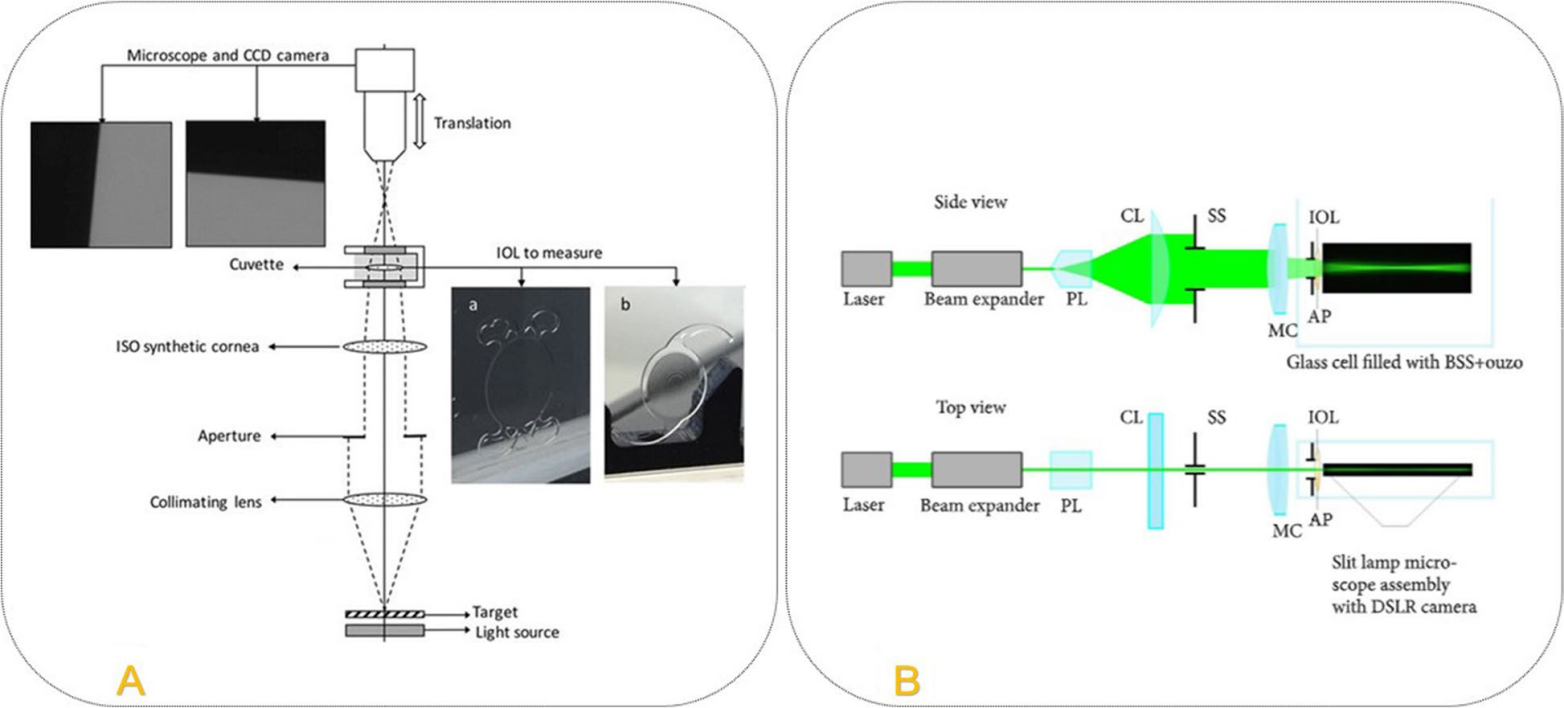
Schematic representation of the custom-made optical setup (**A**) and a sketch of the experimental setup of the laser beam making the light path visible (**B**) adopted from T. Eppig et al. [12, 13]

The USAF measurement setup was adopted from Langenbucher A. [10, 11]. A custom-made optical bench was employed in this study (Fig. 2). It’s an image forming system that can generate images at different focal planes. The 1951 United States Air Force - USAF resolution test chart was used as a test object to study the imaging performance of the IOLs. The USAF target acting as an object is illuminated and a collimating lens is in the pathway of the rays. A collimated light pencil is traced to the synthetic model cornea with spherical aberration of 0.26 microns (ISO 2 model cornea). The samples (IOLs) were placed in a liquid medium inside a cuvette filled with balanced salt solution which is defined in the ISO 11,979 standard. The cuvette was located in the pathway of the converging refracted rays from the synthetic cornea. A charge coupled device (CCD) camera and the objective were mounted on a motorized translation stage (travel range 25 mm, resolution ± 1.25 μm) to adjust the image plane at different vergences. The objective and the camera as a single component scan the range of the focus with step size of ± 2.5 μm axially and obtain images generated by the IOLs at different vergences. The obtained images were analyzed and the image contrast was extracted using matrix laboratory software (MATLAB 2019b).

### Light field

The Light field measurement setup was adopted from S. Reiss et al. and T. Eppig et al. [10, 11]. The setup comprises a monochromatic line light source, an eye model, and an image acquisition system (Fig. 2). The IOL is positioned in a cuvette filled with water mixed with 1 drop of 10% fluorescein to visualize the imaging properties. The lens is arranged in the beam path in such a way that a laser beam passes through it perpendicularly. The course of the rays behind the IOL is made visible due to the fluorescence excitation in the water-fluorescein mixture. The imaging properties of the lens are visualized with a camera (Canon 6D), which is arranged perpendicularly to the beam propagation.

The image acquisition system includes a consumer grade digital single-lens reflex camera and the microscope unit of an ophthalmological slit lamp. A diode-pumped solid-state laser module with a wavelength of 532 nm and a beam diameter of 1.5 mm is used as light source. A reversed beam expander further reduces the laser beam diameter and a Powell lens generates a divergent laser line with homogenous intensity distribution. A cylindrical lens (CL, f = 40 mm) then collimates the laser fan in one dimension and a slit stop (SS, 0.3 mm width) is used to form a rectangular laser line. The eye model’s components are an achromatic doublet (LAO0434, Melles Griot BV, Didam, The Netherlands) serving as model cornea, according to ISO 11979-2:2014 and the intraocular lens under test in a cuvette. An aperture stop (AP, Ø=4.5 mm) is placed directly in front of the IOL in order to simulate a physiological pupil. Positioning of the sample (IOL) within the cuvette is managed with a special IOL holder and the cuvette itself is placed on a 3D-printed customstage.

## Results

The created defect area in the hydrophobic group was slightly deeper and larger than in the hydrophilic group. The Scanning Electron Microscopy revealed dimensions with an average of 80 μm x 80 μm for the hydrophobic lenses and 70 μm x 70 μm for the hydrophilic lenses, respectively.

### USAF

**Fig. 3:**
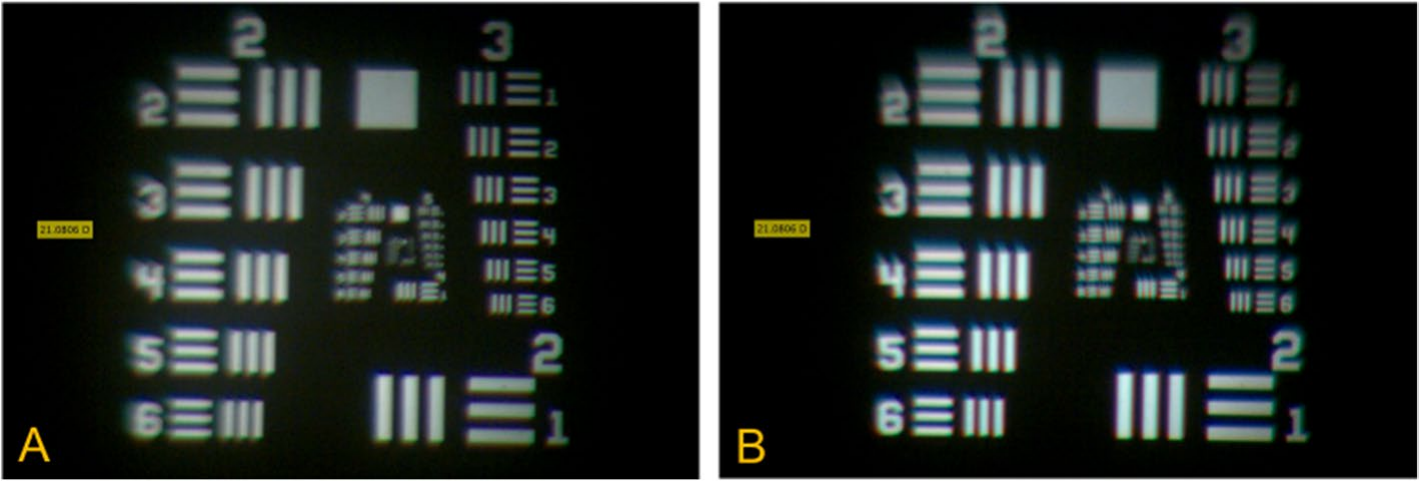
Images of the United States Air Force (USAF) targets at the focal position of the IOL. Left image (**A**) showing a hydrophilic IOL sample in unmodified condition. Right image (**B**) showing the same hydrophilic IOL with tiny defects (YAG-pits, *n* = 5). Note that the image (**B**) is more blurred and lines in group 3 already hard distinguishable

**Fig. 4:**
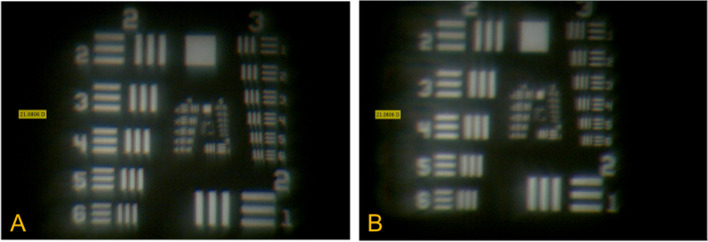
Image of the United States Air Force (USAF) target at the focal position of the IOL. Left image (**A**) showing a hydrophobic IOL sample in unmodified condition. Right image (**B**) showing the same hydrophobic IOL with tiny defects (YAG-pits, *n* = 5). Note the contrast of image (**A**) is higher and lines better distinguishable than in (**B**)

Figure 3 shows the image of the illuminated US Air Force target at focal plane for a 21 D hydrophilic IOL. The direct comparison shows that the unmodified lens produces a sharper image than the lens with the YAG pits. The lens with the defects (*n* = 5) leads to a more blurred image and to the impression of a different contrast with a kind of halo/glare effect (note group 3 and 5 in Fig. 3B). Figure 4 shows the results for the hydrophobic IOL respectively. Again, by comparing these two images it is obvious that the contrast of the first image (note group 2 and 4 of Fig. 4 A) is higher and that the lines are more clearly visible and distinguishable than in the image of the lens with the defects (Fig. 4B). These results confirmed that defects have adverse effects in hydrophilic and hydrophobic IOLs. The lenses with YAG pits (*n* = 5) generated images with lower contrast at the focal position. Moreover, it was shown that the image contrast (at least with this specific laboratory test) was slightly higher in unmodified hydrophilic IOLs compared to unmodified hydrophobic IOLs.

### Light field

**Fig. 5:**
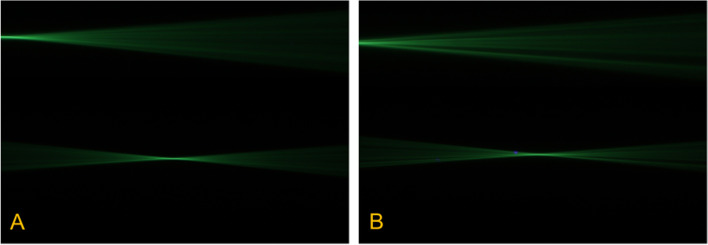
Image showing light propagation. Side view of the light that scattered in the medium containing water and fluorescein. Light beams are reflected from the hydrophilic, unmodified IOL (**A**) in a regularly style, whereas the same IOL with defects (YAG-pits, *n* = 5) showing more scattering and splitting of the beam (**B**)

**Fig. 6:**
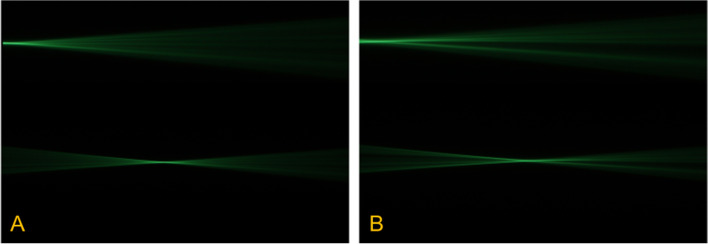
Image showing light propagation. Here, the light beams are reflected from the hydrophobic, unmodified IOL (**A**) in a regularly style, whereas the same IOL with defects (YAG-pits, *n* = 5) showing more scattering and splitting of the beam (**B**). The extent of this effect and impact level is strongly dependent on the position of the defects

The results of the light field measurement setup showed no statistically significant difference between unmodified IOLs and defected ones (Figs. 5 and 6). However, changes were observed when evaluating the light beam (note Figs. 5B and 6B). Hydrophilic and hydrophobic IOLs with YAG-pits led to a kind of dispersion and scattering effect. It has to be emphasized that the extent of this effect and the impact level is strongly dependent on the position of the defects and seemed to be slightly higher in hydrophobic IOLs.

**Fig. 7:**
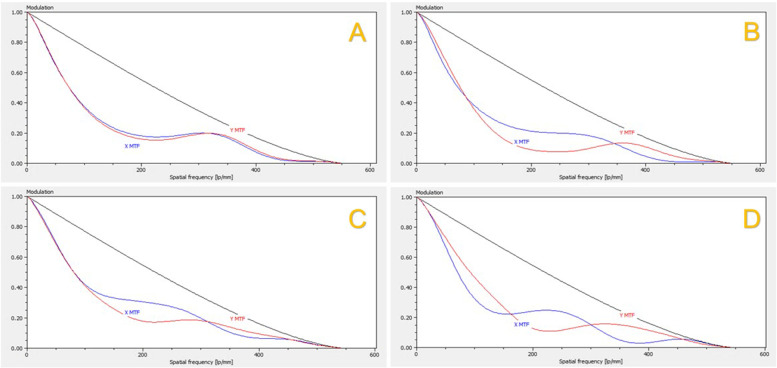
Modulationtransferfunction (MTF) as a measure for the contrast transfer as a function of spatial frequency at focal plane, measured with an aperture of 4.5 mm. Left images showing the MTF curves of the unmodified hydrophilic (**A**) and hydrophobic IOL (**C**) and right images showing the decrease of MTF in the damaged IOLs. **B**: hydrophilic IOL with YAG-pits. **D**: hydrophobic IOL with YAG-pits.

The modulation transfer function (MTF) as a measure for the contrast transfer as a function of spatial frequency at focal plane showed a deterioration in hydrophilic and hydrophobic IOLs with YAG-pits. Again, due to the measuring principle/method, the position of the defect in the IOL is particularly important and determines the extent of the change. The selected aperture size also affects the results. Figure 7 shows the MTF (measured with 4.5 mm aperture) of the unmodified hydrophilic and hydrophobic IOLs compared to the IOLs with the defects. In all cases a decrease of the MTF according to the defects could be shown. In a side study it could be shown that the more YAG-pits were located directly within the measurement zone, the more pronounced were the effects on the MTF.

## Discussion

Another retrospective study of > 3000 cases analyzed PCO formation and YAG-capsulotomy rates in patients, who underwent cataract surgery with either hydrophobic or hydrophilic acrylic intraocular lens implantation. In the 4-year follow up, PCO that required capsulotomies occurred significantly less frequent in patients who had received a hydrophobic IOL (31.57%) compared to hydrophilic IOL implants (56.6%). The study group summarized that hydrophobic lenses seem to be superior regarding both medical and economic results [14]. Considering the high prevalence of cataract, the economic burden associated with adverse effects of cataract extraction and PCO formation is of great relevance.

IOL pitting or IOL damage seems to be a relatively common side effect. In the past, some studies investigating the incidence of laser defects in IOLs came up with a relatively high number of cases. Whereas one study found 11.7% of severe YAG damage, another found up to 19.8% [4]. Different numbers of occurrence could be due to different optical properties of lens models, because the insight during YAG-capsulotomy is different. In addition, various IOL models show different behavior in the capsule. There are differences of dimensions of contact to the posterior capsule due to the individual geometry or angulation of the haptic [15].

Acrylic intraocular lenses with different water content, hydrophilic and hydrophobic, seem to be affected differently by Nd:YAG treatment in terms of wavefront aberrations [16]. The iatrogenic damage to intraocular lenses during YAG laser capsulotomy is caused by inappropriate focusing, acoustic shock waves and heat conduction [17]. These defects in the material of the IOL are called YAG-pits or YAG-shots. A previous study conducted by the author (AFB) already confirmed that there are differences in the defects depending on material properties and water content in acrylic IOLs (hydrophilic vs. hydrophobic) [18]. In their in vitro study microscopy and environmental scanning electron microscopic (ESEM) images were used to visually analyze the defects. Additionally, wavefront measurements were taken for power mapping and Raman spectroscopy was performed. Vertical and horizontal dimensions of the defects were analyzed and compared, and Raman line scans assessed the changes in the chemical structure in the defect area and surrounding area of the IOL. Results showed that Nd:YAG seems to have greater impact on hydrophobic IOL materials as that damage was larger and more frayed than that in hydrophilic materials. Moreover, it was shown that there is a larger and more distinctive damage area in IOLs (with chemical changes in the material) than it is visually recognizable [18]. Another experimental study showed that defects are more severe in rigid materials and less pronounced in soft materials and that shape and form varies greatly depending on the material [12].

The impact of YAG laser capsulotomy on IOL position has also been studied. It was shown that in around 75% of the cases, either decentration, tilt or hyperopic axial displacement (shift) can occur [13]. Another study found that YAG laser capsulotomy performed within one year after cataract surgery lead to significant hyperopic change, in which the anterior chamber depth alteration affects the hyperopic shift significantly [19].

Unfortunately, YAG-pits are quite common in clinical practice. In a survey conducted by the authors among 27 colleagues who regularly perform YAG capsulotomies themselves, it was found that patient incompliance (movements with the head), poor visibility and the time factor were the decisive causes of incorrect focusing during the procedure. It can be assumed that posterior capsulotomy, as a supposedly simple procedure, is often not carried out with the necessary precision. Modern IOL designs try to facilitate a particularly good and close contact of the lens with the posterior capsule contributing to PCO prevention. This fact complicates the capsulotomy and increases the risk of lens damage due to incorrect focusing. YAG capsulotomy should not be considered trivial but should be carried out with precision and without time pressure, just like surgery itself. Several techniques have been described for Nd:YAG capsulotomy. In clinical practice, the “Cruciate Pattern technique” and the “Circular Pattern technique” are the most common procedures. Both techniques have been proved as safe and effective, having certain advantages and disadvantages [20]. The cross like pattern attempts to prevent free-floating parts of the capsule in the vitreous, as the flap retracts out of the visual axis but is still attached. With the circular pattern technique laser spots do not have to be placed within the central optical zone of the IOL. Therefore, the chance of central pitting is lower.

For many years it has been debated whether YAG-pits in IOLs have any impact on the visual acuity or the quality of vision. With this experimental setup, displaying the beam path, we were able to confirm that iatrogenic pits in IOLs do have an influence on the overall quality of the lens and lead to scattered light. It is important to know that the severity level of the changes is dependent on the size, dimension, and position of the defects in the intraocular lens.

As already shown by the authors with previous experimental studies, the visible effects (dimension of the defects) are more pronounced in hydrophobic material. Again, we were able to visualize defects in hydrophobic and hydrophilic intraocular lenses, but it has to be emphasized that in most laboratory measurement methods the extent of the deterioration depends on the position of the defect. Thus, it can be assumed that clinical symptoms are also very different. Many other factors seem to play a role, including the lens model and design, the optical properties of the IOL (material, water content).

More than 34 million cataract procedures are performed per year and patients claim best postoperative results also in routine (standard) cataract surgery [21]. Demands are getting higher and higher and refractive targets are becoming more and more important.

With this laboratory work we want to draw attention to this topic and show that ophthalmologists should take utmost care to prevent inappropriate defocusing and YAG-pits during the procedure. Companies producing laser devices are required to develop innovations in this sector to reduce this possible complication in YAG capsulotomy procedures. This could be achieved with safety features on the laser device that prevent defocus and lens destruction. In addition, additional research work should be done on new IOL materials that are more resistant to YAG-pits to minimize negative effects of iatrogenic defects.

Although it has been found that the effects can vary greatly and laboratory results cannot be extrapolated 1:1 to the clinic, we think that the in vitro results presented here should be taken seriously. YAG-pits do have an influence on overall quality of vision and therefore may decrease patient satisfaction in daily life. More clinical studies evaluating clinical symptoms of YAG-pits have to be performed and the topic should have a higher awareness, as PCO and posterior capsulotomy is a very common procedure all over the world. It should also be evaluated if “premium lenses” (multifocals, enhanced depth of focus IOLs, toric IOLs) and their optics are even more sensitive to such defects than monofocal IOLs.

### Limitations

A limitation of this work is that these images do not directly reflect the reality in the human eye, where all focal points will be superimposed because of different object distances. This experimental study can just provide an insight in the underlying optic principle of various IOLs. Therefore, this method can provide an estimation on the expected amount of effects like halo/glare/straylight and does not correlate exactly with the actual visual effects that might be perceived by a human. It can be assumed that in a real-life scenario inter-individual differences in number, position and size of the defects in the optics can be expected and therefore consequences can vary greatly depending on the individual case. It is not possible to interpret the effects in a general way. Furthermore, only the cruciate pattern was simulated here with cross shaped defects exactly in the center of the optics, further experiments to evaluate the circular pattern are planned. In another study, authors (AFB, EMB) used micro-computed tomography (µCT) technology to analyze the defects in more detail. This work, which is currently under review, should provide further insight to better understand the impact on IOL/materials [22].

## Conclusion

The effect of iatrogenic YAG-pits in intraocular lenses and their negative impact on overall quality of vision including halo, glare, effects under mesopic conditions and influence in daily life is still controversial. Our laboratory study using an experimental setup and analyzing USAF test targets, measuring the light field and the MTF showed that YAG-pits do have an impact on the overall quality of the IOL. The dimension of the deterioration is dependent on the number of defects, size and position of the YAG-pits within the optic. By simulating the cruciate pattern comparing unmodified IOLs and IOLs with 5 tiny defects within the central zone of the optic, we were able to show that YAG-pits are reducing the imaging quality of monofocal IOLs. These defects behave as a new Huygens source and distribute a spherical wave to the wall of pixels of the CCD. Therefore, these spherical waves additionally illuminate the background of the US air force target. It can be assumed that differences in the optical properties, material, water content and refractive index play an important role and affect the results. More studies are needed, including laboratory trials to better identify differences, but also large, multicenter clinical evaluations to better assess symptoms in daily life.

## Data Availability

The authors confirm that the data supporting the findings of this study are available within the article.
